# Robotic transanal minimally invasive surgery (r-TAMIS): perioperative and short-term outcomes for local excision of rectal cancers

**DOI:** 10.1007/s00464-024-10829-4

**Published:** 2024-05-06

**Authors:** Guglielmo Niccolò Piozzi, Ania Przedlacka, Rauand Duhoky, Oroog Ali, Yasser Ghanem, Richard Beable, Antony Higginson, Jim S. Khan

**Affiliations:** 1 Department of Colorectal Surgery, Portsmouth Hospitals University NHS Trust, Portsmouth, UK; 2 Department of General Surgery, Gateshead Health NHS Foundation Trust, Gateshead, UK; 3 Department of General Surgery, Isle of Wight NHS Trust, Newport, UK; 4 Department of Radiology, Portsmouth Hospitals University NHS Trust, Portsmouth, UK; 5 University of Portsmouth, Portsmouth, UK

**Keywords:** Robotic surgery, Rectal cancer, TAMIS, Robotic transanal surgery, Total mesorectal excision

## Abstract

**Background:**

Transanal minimally invasive surgery (TAMIS) is an advanced technique for excision of early rectal cancers. Robotic TAMIS (r-TAMIS) has been introduced as technical improvement and potential alternative to total mesorectal excision (TME) in early rectal cancers and in frail patients. This study reports the perioperative and short-term oncological outcomes of r-TAMIS for local excision of early-stage rectal cancers.

**Methods:**

Retrospective analysis of a prospectively collected r-TAMIS database (July 2021–July 2023). Demographics, clinicopathological features, short-term outcomes, recurrences, and survival were investigated.

**Results:**

Twenty patients were included. Median age and body mass index were 69.5 (62.0–77.7) years and 31.0 (21.0–36.5) kg/m^2^. Male sex was prevalent (*n* = 12, 60.0%). ASA III accounted for 66.7%. Median distance from anal verge was 7.5 (5.0–11.7) cm. Median operation time was 90.0 (60.0–112.5) minutes. Blood loss was minimal. There were no conversions. Median postoperative stay was 2.0 (1.0–3.0) days. Minor and major complication rates were 25.0% and 0%, respectively. Seventeen (85.0%) patients had an adenocarcinoma whilst three patients had an adenoma. R0 rate was 90.0%. Most tumours were pT1 (55.0%), followed by pT2 (25.0%). One patient (5.0%) had a pT3 tumour. Specimen and tumour maximal median diameter were 51.0 (41.0–62.0) mm and 21.5 (17.2–42.0) mm, respectively. Median specimen area was 193.1 (134.3–323.3) cm^2^. Median follow-up was 15.5 (10.0–24.0) months. One patient developed local recurrence (5.0%).

**Conclusions:**

r-TAMIS, with strict postoperative surveillance, is a safe and feasible approach for local excision of early rectal cancer and may have a role in surgically unfit and elderly patients who refuse or cannot undergo TME surgery. Future prospective multicentre large-scale studies are needed to report the long-term oncological outcomes.

The gold standard technique for rectal cancer treatment is total mesorectal excision (TME), which is characterised by the removal of the rectum with surrounding mesorectum without breaching the mesorectal fascia to allow for oncological clearance and reduce the risk of local recurrence [1]. However, TME may be excessive for early-stage local tumours (cT1N0) and can be associated with high morbidity and postoperative functional changes [2–6]. These patients can benefit from a transanal approach. Transanal excision techniques have steadily developed in the last decades from conventional transanal excision (TAE) using anoscopic instruments to transanal endoscopic microsurgery (TEM) with improved surgical field exposure, oncological specimen quality (reduced R0 and tumour fragmentation), recurrence rates, and allowed accessibility to the proximal rectum [7, 8]. However, TEM is characterized by high equipment cost, high complication rate, complex instrumentation, and a steep learning curve, therefore available only in specialized centres with advanced training [9].

The introduction of transanal minimal invasive surgery (TAMIS) has optimized the transanal accessibility to rectal cancers allowing to excise also voluminous tumours even in the upper rectum [10]. TAMIS benefits from the use of standard laparoscopic instruments (no need for a specific platform), the transferability of operating skills from standard laparoscopy, the independency from a lesion-dependent positioning (specific of TEM) leading to faster setup, and use of the lithotomy position with ease of performing an abdominal access in case of need [11]. TAMIS has rapidly gained popularity but has limitations linked to the rigidity of standard straight instruments, which operate in a parallel fashion with poor triangulation, often in conflict with the endoscope inside the confined space of the rectum causing challenging exposure, dissection, and suturing [12]. It is unclear if the adoption of articulated laparoscopic instruments can overcome these limitations [13].

The implementation of the robotic approach to TAMIS (r-TAMIS) could potentially overcome the technical limitations of laparoscopic TAMIS allowing optimization of oncological radicality in narrow spaces through enhanced dexterity and ergonomics, tremor-free motion, and optimized high-definition 3D view. r-TAMIS has been used for less than a decade and its feasibility has been demonstrated in cadaveric models [14] and surgical series on benign and malignant tumours. However, large descriptive series (above ten cases) are still limited, especially on rectal cancers [10, 15–18]. Moreover, small studies comparing laparoscopic to robotic TAMIS found no significant differences other than the total direct cost between the two approaches [19].

The implementation of routine colorectal cancer screening programs with consequent increase of early cancer diagnosis susceptible to local excision through endoscopic techniques (endoscopic mucosal resection or endoscopic submucosal dissection) or surgical procedures (TEM, TAMIS) and the opportunity to have a surgical safe and feasible alternative for patients who are frail or unfit for standard TME has amplified the interest in transanal excisions.

This study reports the perioperative and short-term oncological outcomes of r-TAMIS for early-stage local rectal cancers in a tertiary oncological colorectal referral centre.

## Materials and methods

### Study population

This is a retrospective study evaluating all consecutive patients with early-stage local rectal cancers undergoing r-TAMIS resections from July 2021 to July 2023 in a tertiary colorectal referral centre with expertise in robotic surgery. The patients were prospectively enrolled in a dedicated database. Institutional Review Board approved the study (IRAS ID 293129). All patients provided informed consent for research studies.

Primary aim was to report the perioperative outcomes of r-TAMIS for early-stage local rectal cancers. Secondary aim was to access short-term oncological outcomes.

Indications for r-TAMIS and inclusion criteria were: (1) cT1N0 rectal adenocarcinomas based on rectal magnetic resonance imaging (MRI) or endorectal ultrasound (ERUS) staging; (2) cT2N0 but declined/unfit for major resection/stoma; (3) rectal lesions with high grade features suspicious for cancer and thus not suitable for endoscopic or conventional resection; (4) robotic approach; (5) elective setting; (6) curative surgery; (7) age above 18-year-old. Exclusion criteria: (1) palliative surgery; (2) stage IV.

Distance between tumour’s caudal edge and anorectal junction was assessed via digital rectal examination and rectal MRI. Clinical staging was performed via colonoscopy with biopsy, thoracic/abdominopelvic computed tomography (CT), rectal MRI, and ERUS.

All patients were discussed at a multidisciplinary (MDT) meeting for treatment strategy. Neoadjuvant chemoradiotherapy (nCRT), either short- or long-course, was given at discretion of MDT in case of T2 or T3 tumours.

The short-course protocol involved 25 Gy in five fractions over 5 weekdays whilst the long-course involved 45–50 Gy in 25 fractions over 5 weeks with concomitant chemotherapy (capecitabine). Clinical re-staging was performed at six weeks after nCRT completion.

Pathological staging was provided according to the American Joint Committee on Cancer (AJCC) 8th edition staging system [20] during data review.

Complications were assessed according to Clavien-Dindo’s classification [21]. Conversion was defined as the need to change to a different transanal or transabdominal approach. Adjuvant chemotherapy protocol followed international guidelines.

Post-operative follow-up protocol included pelvic MRI and flexible sigmoidoscopy every three months, for year 1 and 2. Colonoscopy and CT-CAP (CT-chest-abdomen-pelvis) at 1-year mark, and one CT-CAP at the 2-year mark. In years 3 and 4, they received pelvic MRI every six months, along with a flexible sigmoidoscopy. Finally, in year 5 they receive a colonoscopy and CT-CAP.

Overall survival (OS) was measured from date of surgery to date of death/last follow-up, disease-free survival (DFS) to date of tumour recurrence. Recurrence was diagnosed through endoscopic or radiological detection of enlarging lesions or by histological confirmation. This study follows the STROBE statement for cohort studies [22].

### Surgical technique

**Fig. 1:**
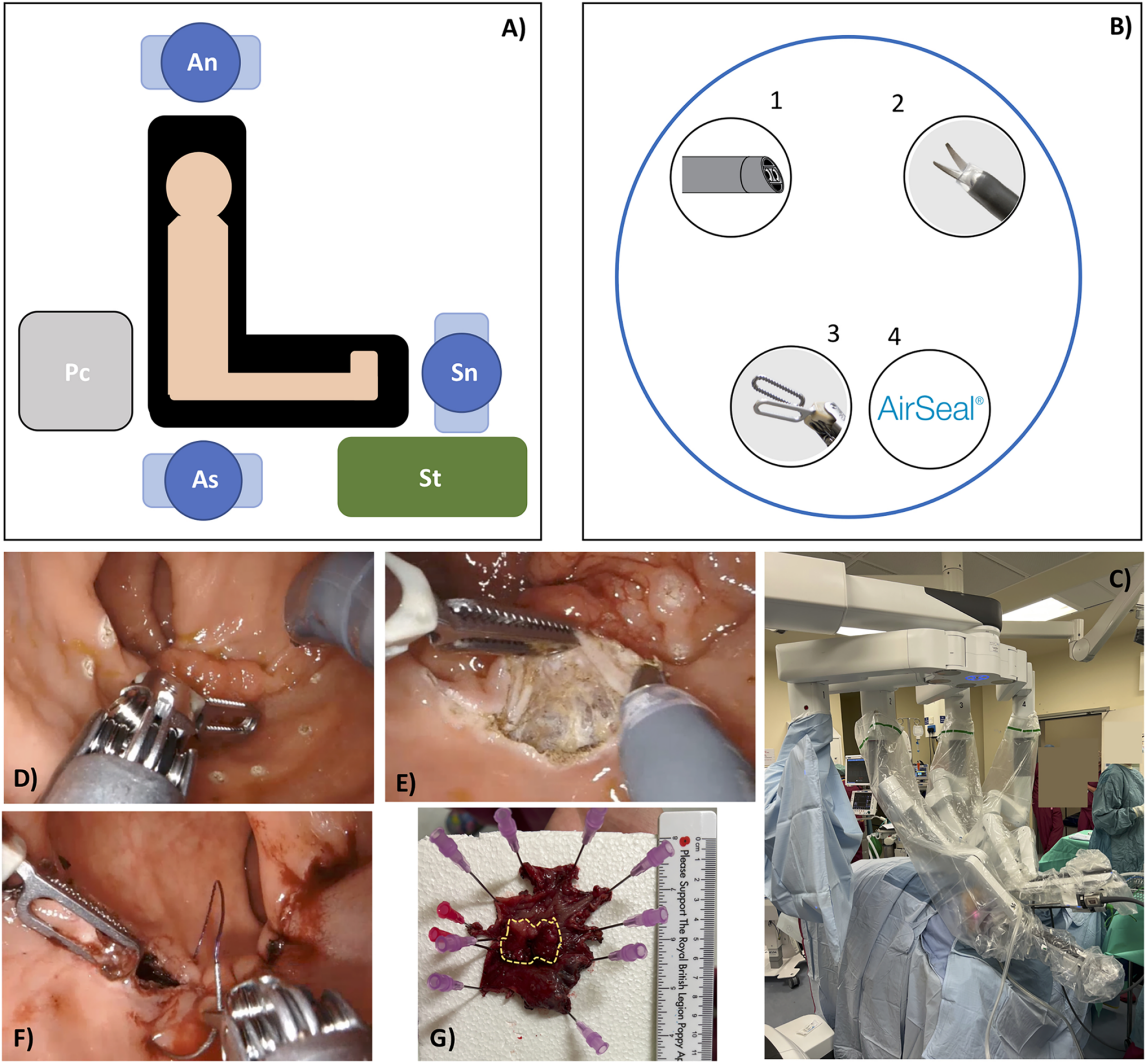
**A** Scheme of patient positioning and theatre configuration; An: anesthetist: As: assistant surgeon; Pc: patient cart; Sn: scrub nurse; St: scrub nurse table. **B** Port placement through a GelPOINT. **C** Patient cart docking position. **D**, **E**, **F** Surgical steps. **G** Specimen on oriented and pinned on a polystyrene sheet before fixation (yellow dotted line shows the tumor margin)

All patients underwent preoperative mechanical bowel preparation [23]. Prophylactic antibiotic was administered at induction. Scopolamine butylbromide (20 mg) was administered at the start of the procedure. The Da Vinci Xi (Intuitive Surgical, Inc., Sunnyvale, CA, USA) platform was used for all patients with three arms setting. The patient was positioned in a modified lithotomy position during 2021. From January 2022 the patient was positioned on the left side with hips flexed to 90 degrees to achieve adequate exposure. A GelPOINT Path Transanal Access Platform (Applied Medical, Rancho Santa Margarita, CA, USA) was used. Three 8 mm robotic ports and an AirSeal (Intelligent Flow System, ConMed, Utica, NY, USA) port were positioned on the GelPoint platform before inserting it in the anus. Ports 1 and 2 are placed in the left and right upper quadrants of the GelPoint, almost skirting the outside plastic ring (Fig. 1B). Port 3 and the Airseal port are placed closer to midline in the left and right lower quadrants (Fig. 1B). The platform is secured with silk sutures. The DaVinci Xi System is docked from the patient’s right side with the upper abdomen setting (Fig. 1A, C). Bipolar fenestrated forceps are placed in port 3, the camera in port 1 and monopolar curved scissors in port 2. Arm 1 is not docked; however, it remains draped to ensure sterility of the surgical field (Fig. 1C). SynchroSeal (Intuitive Surgical, Inc., Sunnyvale, CA, USA) can later replace the bipolar forceps in case of voluminous, vascularized, and higher lesion with difficult access needing advanced haemostasis. This instrument is especially useful for lateral lesions which are usually more vascularized and at risk of bleeding. The robotic needle driver is used in place of the monopolar scissors for closing the wall defect. Pneumorectum is created at 8 mmHg through the AirSeal port, which is used to minimise rectal ballooning and camera fogging and is additionally utilised as the assistant port.

The procedure begins by the assessment of the anatomy of the rectal cancer. The circumference of the lesions is marked with the monopolar scissors with a 1 cm margin to ensure adequate excision (Fig. 1D). A full thickness dissection is performed to the mesorectum with the aid of monopolar scissors and bipolar forceps (Fig. 1E). The assistant provides evacuation of smoke and suction to optimise the view. Once the lesion is completely dissected, irrigation and adequate haemostasis is performed. The rectal defect is routinely closed with 3/0 absorbable barbed sutures (Fig. 1G). The surgical technique was previously reported [24].

Patients are routinely admitted overnight for monitoring and discharged the following day with stool softeners and a five-day course of oral antibiotics.

### Specimen delivery to pathologist

All specimens were oriented and pinned on a polystyrene sheet before fixation and sent to pathology to facilitate an oriented histopathologic evaluation of the specimen.

### Statistical analysis

Patient characteristics were summarized using basic descriptive statistics. Continuous variables were presented as median (interquartile range, IQR) or mean ± standard deviation accordingly. Categorical variables were expressed as proportions. Statistical analysis was performed using IBM SPSS Statistics for Macintosh, version 28 (IBM Corp., Armonk, NY, USA). Survival and recurrence rates were estimated through Kaplan–Meier model. Confidence intervals were estimated at 95%, and significance level was set at *p* = 0.05.

## Results

### Patient characteristics

Twenty patients underwent r-TAMIS during the study period. Characteristics of patients and primary tumours are listed in Table 1. Median age was 69.5 (62.0–77.7) years. Most patients were male (*n* = 12, 60.0%). Median body mass index (BMI) was 31.0 (21.0–36.5) kg/m^2^. Median Charlson Comorbidity Index was 5.0 (4.0–5.0) ranging from 3 to 9. ASA III was predominant (66.7%). Only two patients (10.0%) had previous abdominal surgery. Most patients had a primary rectal tumour (*n* = 19, 95.0%). Median distance from AV was 7.5 (5.0–11.7) cm. Median maximal diameter was 25.0 (20.0–34.0) mm. Tumour location was mostly posterior and postero-lateral (35.0 and 25.0%, respectively). Most tumours were cT2 (45.0%) followed by cT1 (35.0%). All patients were cN0. Two patients (10.0%) underwent nCRT (one short- and one long-course). Indications to r-TAMIS are described in Table 2.

**Table 1:** Series characteristics

	*n* = 20
Age, years	69.5 (62.0–77.7)
Male sex	12 (57.1%)
BMI, Kg/m^2^	31.0 (21.0–36.5)
Charlson Comorbidity Index	5.0 (4.0–5.0)
ASA	
II	6 (28.6%)
III	14 (66.7%)
Tumour	
Primary	19 (95.0)
Recurrence	1 (5%)
Distance from AV, cm	7.5 (5.0–11.7)
Tumour diameter at staging, mm	25.0 (20.0–34.0)
Tumour location	
Anterior	2 (10.0%)
Antero-lateral	3 (15.0%)
Posterior	7 (35.0%)
Postero-lateral	5 (25.0%)
Lateral	3 (15.0%)
cT	
cTx	2 (10.0%)
cT0	2 (10.0%)
cT1	7 (35.0%)
cT2	9 (45.0%)
cN0	20 (100.0%)
Previous surgery	2 (10.0%)
nCRT	2 (10.0%)

Values are presented as number (percentage) or median (interquartile range, IQR)

*ASA* American Society of Anaesthesiologists’ score, *AV* anal verge, *BMI* body mass index, *nCRT* neoadjuvant chemoradiation

**Table 2:** Surgical and postoperative outcomes

	*n* = 20
Indication	
Unsuccessful EMR/ESD	2 (10.0%)
Major comorbidities/old age	5 (25.0%)
Refusal of major surgery/stoma	7 (35.0%)
Tumour characteristics	6 (30.0%)
Operation time, min	90.0 (60.0–112.5)
EBL, ml	0.00 (0.00–12.5)
Patient positioning	
Modified lithotomy	4 (20%)
Left lateral	16 (80%)
Conversion, *n*	0 (0%)
Postoperative stay, days	2.0 (1.0–3.0)
Readmission, *n*	2 (10.0%)
Reoperation, *n*	0 (0%)
Minor complications (Clavien-Dindo I-II), *n*	5 (25.0%)
Major complications (Clavien-Dindo III-V), *n*	0 (0%)

Values are presented as number (percentage) or median (interquartile range, IQR)

*EBL* estimated blood loss, *EMR* endoscopic mucosal resection, *ESD* endoscopic submucosal dissection

### Operative outcomes

Median operation time was 90.0 (60.0–112.5) minutes. Median estimated blood loss was 0.00 (0.00–12.5) ml (Table 2). Patient positioning was modified lithotomy for four (20%) patients and left lateral for sixteen (80%) patients. No conversion to trans-abdominal or other transanal procedure were performed. All rectal defects were routinely closed with 3/0 v-lock suture. All procedures were performed fully robotically with no intraoperative complications.

### Postoperative course

Median postoperative stay was 2.0 (1.0–3.0) days (Table 2). Five (25.0%) patients developed postoperative complications in the following 30 days, all graded as I-II according to Clavien-Dindo: minor bleeding (*n* = 2), nausea/bloating (*n* = 1), fever (*n* = 1), and urinary retention (*n* = 1). Two patients (10.0%) were readmitted after developing rectal bleeding eight days after surgery and were treated with observation only successfully. No peritoneal entry was reported. Thirty-day mortality was nil.

### Pathological results

Seventeen (85.0%) patients had an adenocarcinoma whilst three had an adenoma (two with low grade and one with high grade dysplasia; Table 3). R0 resection was achieved in eighteen patients (90.0%). Most tumours were pT1 (55.0%), followed by pT2 (25.0%). Only one patient (5.0%) had a pT3 tumour. Specimen maximal median diameter was 51.0 (41.0–62.0) mm with a tumour maximal median diameter of 21.5 (17.2–42.0) mm. Median specimen area was 193.1 (134.3–323.3) cm^2^ ranging between 17.6 and 500.5 cm^2^. Tumour margins were adequate in all patients apart from two (one pT3Nx and one pT2Nx) having the deep margin involved (< 1 mm). All specimens were retrieved without fragmentation.

**Table 3:** Pathological results and oncological outcomes

	*n* = 20
pT stage	
pT0	3 (15.0%)
pT1	11 (55.0%)
pT2	5 (25.0%)
pT3	1 (5.0%)
Adenocarcinoma	17 (85.0%)
Adenoma	3 (15.0%)
R0 resection	18 (90.0%)
Specimen max diameter, mm	51.0 (41.0–62.0)
Specimen median area, cm^2^	193.1 (134.3–323.3)
Tumor max diameter, mm	21.5 (17.2–42.0)
Margin peripheral, mm	7.2 (5.0–9.7)
Margin deep, mm	4.0 (2.6–5.0)
Adjuvant CRT, *n*	3 (15.0%)
Follow-up, months	11.5 (3.5–17.0)
Mortality	0 (0.0%)
Malign recurrence, *n*	2 (10.0%)

Values are presented as number (percentage) or median (interquartile range, IQR)

*CRT* chemoradiotherapy

### Oncological outcomes

**Fig. 2:**
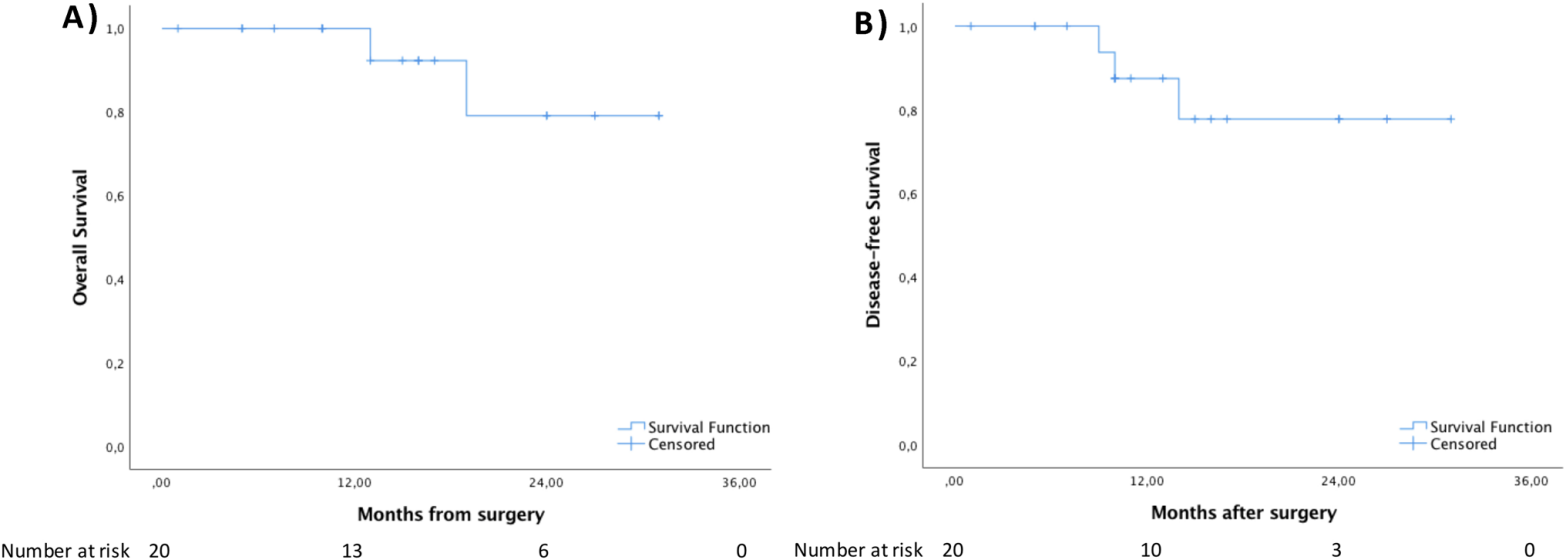
Kaplan–Meier survival curves for **A** overall survival and **B** disease-free survival of r-TAMIS

Median follow-up was 15.5 (10.0–24.0) months (Table 3). During follow-up one patient developed a high rectovaginal fistula five months after the r-TAMIS on the resection bed. Overall survival was 100.0% and 79.1% at 1- and 3-years, respectively (Fig. 2A). Disease-free survival was 87.5% and 77.8% at 1- and 3-years, respectively (Fig. 2B). The patient who had a poorly differentiated pT3Nx tumour with shortest peripheral margin of 4.5 mm and deepest margin of 4.0 mm developed a local recurrence after ten months which would have required an abdominoperineal resection, however the patient declined surgery. One patient (pT2) developed a liver recurrence after nine months and is receiving chemotherapy (oxaliplatin and capecitabine). One patient (pT2) developed multiple liver and lung recurrence 14 months after primary surgery and underwent palliative treatment with FOLFIRI before exitus at 19 months. One patient developed a benign recurrence after eleven months (previous tumour was a tubular adenoma with low grade dysplasia) for which a redo r-TAMIS was performed.

## Discussion

Robotic TAMIS, with strict postoperative surveillance, is a safe and feasible approach with excellent perioperative outcomes for even surgically unfit and elderly patients who refuse or cannot undergo traditional radical resection (i.e., TME) due to frailty.

Gold standard of rectal cancer treatment remain TME surgery, which allows for an embryologically driven resection of the rectum together with its mesorectal envelope. However, the risk of lymphatic metastasis differs according to tumor biology and T staging, being up to 8.6% for pT1 tumours [25, 26]. Significant predictors of lymph node metastasis for pT1 tumours are sm3 (involvement of the lower third of the submucosa), lymphovascular invasion, and location in the rectum lower third [25, 26]. Therefore, endoscopic resection could be indicated as alternative to TME for selected low risk pT1 tumours.

The 2023 National Comprehensive Cancer Network (NCCN) Rectal Cancer guidelines [27] indicate transanal local excision (i.e., TEM/TAMIS) to be appropriate only for cT1N0, mobile, nonfixed, ≤ 8 cm from AV, < 30% bowel circumference, < 3 cm in size rectal tumors with pathological report showing clear margins (> 3 mm), negative lymphovascular and perineural invasion and well-moderate differentiation. Standard TME is recommended in case of adverse features [25, 26].

There is limited data on long-term oncological outcomes from high-risk T1 or T2 tumours undergoing local excision [28]. Local excision (only) may provide a high-risk of local recurrence in these patients [29] which could be reduced with adjuvant CRT (for pT1) or completion TME. The ACOSOG Z6041 randomized controlled trial showed that nCRT (capecitabine/oxaliplatin) followed by local excision may be a safe alternative to TME in patients with T2N0 distal rectal cancer [30]. Furthermore, a meta-analysis from Shaikh et al. interestingly reported no statistical difference in local recurrence, overall survival, and disease-free survival between patients undergoing nCRT + local excision and nCRT + TME, stating that the former could potentially be a safe alternative for patients with any T- or N-stage rectal cancer refusing or being unfit for TME [31]. However, this should be evaluated carefully in further clinical trials. The main limitation of local excision is the complete/partial absence of pathological nodal staging which could be responsible for local recurrence as reported in several retrospective studies comparing pT1 rectal cancers undergoing either transanal local excision or radical resection [28, 32, 33]. Careful evaluation of patients with cT1N0 rectal cancers is needed and TME should be indicated in case a pT2 or high-risk features are evident at final pathological examination [27].

Transanal local excision has clear advantages over TME which should be considered for patients with early tumours or unfit for surgery.

r-TAMIS allows for a short operation time (90.0 (60.0–112.5) minutes in the present series), which is associated with lower complication rates, reduced surgical team fatigue, and shorter anaesthesia duration [34]. r-TAMIS does not require a head-down tilt lithotomy position, which is needed especially during minimally invasive TMEs and could be responsible for significant hemodynamic effects on the central nervous system and limbs, worsened by longer operation time [35, 36]. For this reason, the authors believe that a left lateral or a prone position could be beneficial.

r-TAMIS allowed for the excision of voluminous specimens (193.1 (134.3–323.3) cm^2^) with a diameter ranging up to 91 mm. Moreover, median tumour dimension was 21.5 (17.2–42.0) mm with a maximal value of 58 mm. In the present series 90% of patients had an R0 resection with two patients having a deep margin involved (< 1 mm). This shows that extending the indication beyond the 3 cm limit indicated by the NCCN guidelines [27] can be considered safe as proposed by other studies, therefore, tumour dimension should not necessarily be considered a contraindication [10].

The NCCN guidelines also limit the indication for tumours up to 8 cm from the AV. r-TAMIS allows for extending the height of dissection as shown in the current study with a median distance from the AV of 7.5 (5.0–11.7) cm and a maximal height of 15 cm. This allows the surgeon to expand the indication of transanal excision to the upper rectum and sigmoid which could further benefit from the use of endoscopic robotic platforms as the Flex® Colorectal Drive Robotic System (Medrobotics Corporation, Raynham, MA, USA) [10, 37].

Moreover, since r-TAMIS is a more conservative approach it is associated with low rates of perioperative morbidity with a 25.0% rate, all Clavien-Dindo I-II as reported in the current study. This rate is higher than published reports, which shows a complication rate ranging between 7 and 18.4% for laparoscopic TAMIS [38, 39] and estimated around 10.5% for r-TAMIS [10], but it should be noticed the high rate of unfit patients (ASAIII 66.7%) and the highest BMI reported (31.0 (21.0–36.5) Kg/m^2^) until now for r-TAMIS in the present series. Also, minor postoperative complications are subject to a high level of reporting bias due to their nature.

Postoperative stay for r-TAMIS is short with a median of 2.0 (1.0–3.0) days, compared with current literature [10]. This allows the patient to return to home and to daily routine in a short time which is a relevant factor especially for older and frail patients.

Lastly, no conversion to transabdominal TME or other transanal procedures occurred in the present series showing the safety to complete a TAMIS robotically. However, Jakobsen et al. reported a conversion rate of 4.3% [10]. All these reasons point out the exceptional role of r-TAMIS as a technically safe alternative to standard TME for early rectal cancer, in the short-term perioperative outcomes, especially for frail patients. In the present series only 25% of cases underwent r-TAMIS specifically because of tumour characteristics following NCCN indication criteria [27]. Most patients underwent r-TAMIS for refusal of major surgery/stoma (35.0%) or major comorbidities/age (25.0%) (Table 2). It is critical to carefully evaluate the clinical conditions and patients’ desire before defining surgical strategy as this should be patient and not surgeon tailored. Therefore, TAMIS should be part of the standard armamentarium for an oncological colorectal surgeon and the robotic platform can make it more accessible. This is relevant especially when considering that TME can be characterized by permanent or temporary stoma and is associated to severe morbidity and potentially mortality due to complications including anastomotic leakage, bleeding, sepsis, and urinary/sexual/bowel disfunction which could affect quality of life [2–6].

Compared to laparoscopic TAMIS, the robotic platform allows to better maintain the pneumorectum due to the lower torque force at the ports [17], optimizes the excision depth, and simplifies the suturing and closure of the defect [19]. The use of an AirSeal port further increases the pneumorectum stabilization aiding the surgeon [40].

r-TAMIS benefits the surgeon’s (and assistant) comfort by relocating the surgical team away from between the legs of the patient where the limited space, especially in high BMI patients (as shown in the present series), and instrument clashing could cause discomfort during the surgical procedure [18].

Patient ideal positioning during r-TAMIS is debated and currently under evaluation. The lithotomy position is the most familiar to colorectal surgeons and theatre teams and it allows quick access to the abdomen if needed, however, obturator nerve injury or lower limb neuropathy may occur during overt external rotation [15] and there could be an increased clashing of the robotic arms with the patients’ legs. The prone jack-knife position has a lower occurrence rate of nerve injury, allows for an easier docking with broader robotic arm range of motion [12], however, the patient’s oxygenation and respiration could be of major concerns in an unexperienced team [41]. Despite this, patient position depends on the surgeon’s experience with no difference in clinical outcomes [15]. Furthermore, Tomassi et al. reported a hockey stick decubitus position with parallel docking of the robot providing a large range of motion for the extracorporeal robotic arms [16].

All r-TAMIS were performed with the da Vinci Xi platform in the present series. This platform is superior to the Si and X because of the presence of the boom which facilitates the positioning of the arms in the transanal port optimizing the external positioning and reducing the instrument clashing as much as possible.

Kajmolli et al. compared the Si and Xi platforms reporting three main technical differences: (a) the Si vertically-mounted arms design require the patient to be positioned in an uncomfortable position with asymmetrical hip flexion as opposed to the Xi boom-mounted horizontal arm design, (b) the Si patient cart arms are more bulky compared to the Xi (28 cm vs 19 cm in circumference) which decreases manoeuvrability between patient’s legs, and (c) the abduction pattern of movement of the Si arms could potentially increase the risk of external collision with the patient's legs as opposed to the Xi jack-knife pattern of movement [42].

Despite technical differences between Si and Xi, Yao et al. did not find any statistical difference in operative time, estimated blood loss, length of hospitalization, or pathological outcomes between the two platforms [18].

The da Vinci Single-Port (SP) platform is optimized for transanal excisions and could be beneficial for r-TAMIS [43], although the Xi limitations due to external collision can be resolved by adopting a left lateral position as in the present series or a prone jack-knife position [12].

Currently the literature reports r-TAMIS performed only with the da Vinci robotic platform (S, Si, X, Xi, and SP) or with the Flex® Colorectal Drive Robotic System (*n* = 10 patients) [10, 37]. It will be interesting to see if the new incoming robotic surgical and endoscopic platforms will positively contribute to expanding r-TAMIS indications and feasibility.

r-TAMIS could be cost-effective compared to standard TME for complex cases where the second is not indicated. Two studies have reported the cost of r-TAMIS. Lee et al. reported a median direct cost of 4,440.92$, while Ruiz et al. reported the total expense in materials, i.e., robotic instruments/transanal port, to be 1,889$ per procedure. However, the cost is superior to laparoscopic TAMIS with an increase ranging between 878.9 and 2000$ [15, 19, 44].

Finally, r-TAMIS could have a relatively lower learning curve and shorter procedure times than endoscopic submucosal dissection (ESD) which requires skilled endoscopists, however, the literature is scarce and inconsistent [45, 46]. The results from the multicentric TRIASSIC randomised controlled study comparing ESD to TAMIS are highly awaited [47].

This study has some limitations. First, it is a retrospective study with relatively small numbers possibly suffering from patient selection bias. Second, all procedures were performed by an experienced robotic surgeon and generalizability to other institutions or surgeons with less robotic experience may be limited. Third, this study specifically aims to evaluate the short-term outcomes with a limited follow-up up to 24 months and the functional outcomes were not included in the present study. Finally, no comparison was done with other approaches (standard TME or laparoscopic TAMIS).

This study has several strengths. First, to the authors knowledge this is the first report on r-TAMIS in the UK and shows a significant series considering the short study period of only two years. Second, the surgical procedures were all performed in a standardized fashion following our institutional protocol with a strict follow-up surveillance. Third, this study shows how r-TAMIS can be a feasible approach for frail, high BMI, unfit patients with good perioperative outcomes.

r-TAMIS is still in its infancy with limited data from small series from different institutions using various platforms, positioning, and approaches. Jakobsen et al. [10] reported the r-TAMIS at the end of the stage 2b, i.e., at the exploration level, according to the IDEAL framework [48]. Future prospective multicentre large-scale studies, possibly with a comparator arm (i.e., laparoscopic TAMIS), are needed to report long-term outcomes and further explore the indication criteria for r-TAMIS.

## Conclusion

r-TAMIS appears to be feasible and safe option for transanal excision of early rectal cancer and in surgically unfit and elderly patients who refuse or cannot undergo TME. The ergonomic advantage can help the surgeon with better vision and suturing capability. Future prospective multicentre large-scale studies are needed to report the long-term oncological outcomes.

## Data Availability

The datasets used or analysed during the current study are available from the corresponding author upon reasonable request.
